# Electromyographic Comparison of Forearm Muscle Movements for Fine Skin Suturing Between an Enlarged Pen Needle Holder and a Webster Needle Holder

**Published:** 2013-05-06

**Authors:** Erika Ohata, Kiyoshi Matsuo, Ryokuya Ban, Masato Shiba, Yoshichika Yasunaga

**Affiliations:** Department of Plastic and Reconstructive Surgery, Shinshu University School of Medicine, Matsumoto, Japan

## Abstract

**Background:** For surgical suturing, a Webster needle holder uses wrist supinating with supinator and extrinsic muscles, whereas a pen needle holder uses finger twisting with intrinsic and extrinsic muscles. Because the latter is better suited to microsurgery, which requires fine suturing with less forearm muscle movement, we have recently adopted an enlarged pen needle holder scaled from a micro needle holder for fine skin suturing. In this study, we assessed whether the enlarged pen needle holder reduced forearm muscle movement during fine skin suturing as compared with the Webster needle holder. **Methods:** A fine skin-suturing task was performed using pen holding with the enlarged micro needle holder or scissor holding with the Webster needle holder by 9 experienced and 6 inexperienced microsurgeons. The task lasted for 60 seconds and was randomly performed 3 times for each method. Forearm flexor and extensor muscular activities were evaluated by surface electromyography. **Results:** The enlarged pen needle holder method required significantly less forearm muscle movement for experienced microsurgeons despite it being their first time using the instrument. There was no significant difference between 2 methods for inexperienced microsurgeons. **Conclusions:** Experienced microsurgeons conserved forearm muscle movement by finger twisting in fine skin suturing with the enlarged pen needle holder. Inexperienced microsurgeons may benefit from the enlarged pen needle holder, even for fine skin suturing, to develop their internal acquisition model of the dynamics of finger twisting.

The needle holders frequently used in plastic surgery can be roughly classified as Webster type or pen type. For suturing, the Webster needle holder mainly uses wrist supinating between the radius and ulna with supinator and extrinsic muscles, much like handling a fork, and is the most standard instrument generally used in plastic surgery. On the contrary, the pen needle holder, which is an essential instrument in microsurgery, mainly uses finger twisting between the thumb and index finger, as well as the middle finger, with intrinsic and extrinsic muscles, much like handling a pen or chopsticks.^1^^,^^2^ For this reason, ophthalmologists use the Castroviejo needle holder with its flat handle in ophthalmic plastic surgery, and both plastic surgeons and ophthalmologists use a small pen needle holder with a rounded handle in microsurgery, the design of which is suited to smooth and fine suturing because of easy finger twisting less forearm muscle movement. On the basis of this, we have recently adopted a pen needle holder accurately enlarged from a micro needle holder that permits easy rotation of the rounded handle by twisting the fingers^3^ for cleft lip and palate repair and blepharoplasty.

This study assessed whether the enlarged pen needle holder reduced forearm muscle movement in fine skin suturing as measured by surface electromyography (sEMG), which is an established indicator of muscle movements.^4^

## MATERIALS AND METHODS

### Subjects

This study enrolled 15 subjects (4 women and 11 men; average age: 31.6 ± 6.3 years), consisting of 9 experienced microsurgeons (more than 7 years of experience; mean: 11.7 ± 4.7 years) and 6 inexperienced microsurgeons. All participants were right-handed and had no history of any type of muscular or neurological disease. All subjects gave their informed consent for participation in this study after reading the experimental protocol. This study was approved by our institutional ethics committee for human subjects.

### Suturing protocol

**Figure 1:**
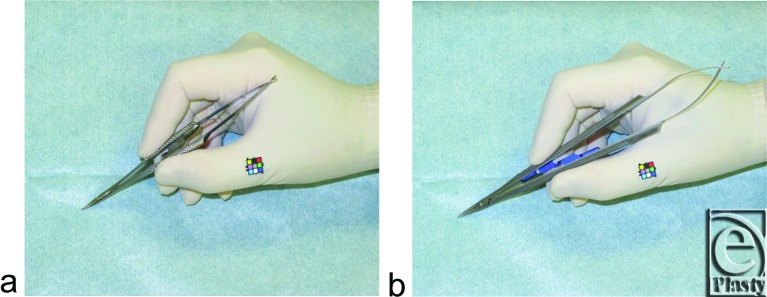
A micro needle holder (*a*) and a newly developed pen needle holder enlarged from a micro needle holder (*b*).

The Webster needle holder (Keisei Medical Industrial Co, Ltd, Tokyo, Japan) and the newly developed enlarged pen needle holder 19 cm in length, scaled from a micro pen holder using a computer-aided design system (EMI Factory Co, Ltd, Miyotamachi, Japan), were adopted in this study (Fig 1). Subjects sutured chicken skin using 5-0 monofilament nylon of 50 cm in length. The semicircular needle employed was 13-mm long with a single radius of curvature measuring 135°, which was similar to those used in closure of thin planar structures, such as skin and vessels.^5^

### Tasks

**Figure 2:**
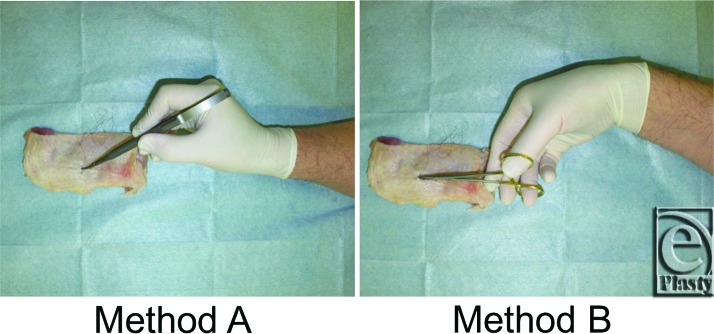
The fine skin-suturing method. Subjects performed fine-skin suturing tasks with the use of an enlarged micro needle holder with pen holding (method A) or a Webster needle holder with scissor holding (method B).

We designed the fine skin-suturing task to include the needle penetrating the skin and being removed from the skin (Fig 2). Surgeons completed this task using 2 methods based on needle holder position: method A used the enlarged micro needle holder with pen holding and method B used the Webster needle holder with scissor holding.

The subjects were instructed to perform the skin-suturing method in random order, taking bites of 2 mm in width from the wound edge, until both methods were repeated 3 times (Table 1).

### Surface electromyography

When skin suturing, surgeons generally move the wrist using palmar flexion, dorsiflexion, and supination, as well as with pronation of the forearm, by the contraction of several forearm muscles.^6^ Because the main muscles for supination and pronation of the forearm are the pronator teres, pronator quadratus, supinator, and biceps brachii, most of which are located in the deep muscle layer, it would have been unfeasible to record the activity of supination and pronation by these forearm muscles using sEMG. Hence, we evaluated wrist movement during surgery by means of the muscle activity involved in palmar flexion and dorsiflexion. The main muscles used in palmar flexion are the flexor carpi radialis, palmaris longus, and flexor carpi ulnaris (FCU), the last of which has the largest tension fraction. Meanwhile, the primary muscles involved in dorsiflexion are the extensor carpi radialis longus, extensor carpi radialis brevis, and extensor carpi ulnaris (ECU), whereby the ECU has the largest tension fraction.^7^

**Figure 3:**
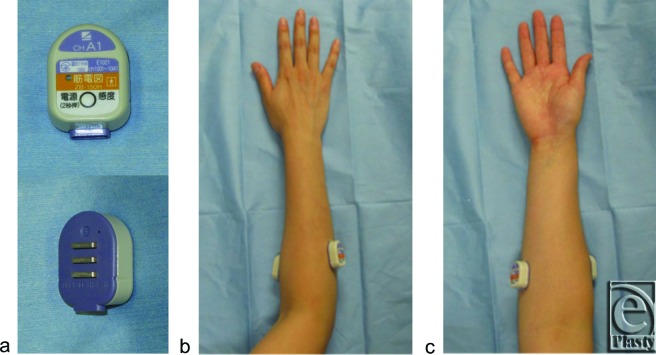
The compact electrode telemeters (*a*) were placed on the skin of the centers of the extensor carpi ulnaris (*b*) and flexor carpi ulnaris (*c*).

**Figure 4:**
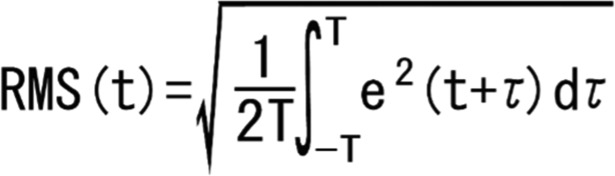
RMS Formula. “e(t)” is the value of electromyographic signal and “(−T, T)” is the interval time calculated.

The compact electrode telemeter consisted of active, reference, and ground electrodes and a wireless transmitter (ZB-150H, Nihon Kohden, Tokyo, Japan), which sent noiseless sEMG data to the host computer for real-time display and storage (WEB-1000, Nihon Kohden, Tokyo, Japan). The distance between the active and reference electrodes was 10 mm, and the ground electrode was located in the center between the other 2 electrodes. The electrode telemeters were placed on the skin over the centers of the FCU and ECU (Fig 3) and secured with double-sided tape on the measuring sites after skin degreasing with alcohol. The sEMG signals were filtered with a 5-Hz low-frequency cutoff and 500-Hz high-frequency cutoff, recorded at a sampling rate of 1000 Hz, and then saved to a personal computer. We subsequently extracted the sEMG signals taken while handling the needle (from penetrating the skin to removing the needle from the skin) and statistically analyzed root mean square (RMS) values (Fig 4). The RMS value of a sEMG signal is associated with the force exerted by the muscle^8^ and is the best way to interpret signals mathematically because the sEMG signal is essentially an alternating microvoltage value at some frequency.^9^

The RMS values for the FCU and ECU sEMG data obtained over the 3 trials for each method were individually averaged for each subject. The sums of the averaged FCU and ECU RMS values were then used as indicators of forearm muscle activity.

### Statistical analysis

The differences in the sums of the averaged RMS values were statistically compared for experienced and inexperienced surgeons using the Wilcoxon signed-rank test with SPSS 20.0 (IBM, Armonk, NY). A *P* < .05 was used as the cutoff for statistical significance.

## RESULTS

### Surface electromyography measurements

**Figure 5:**
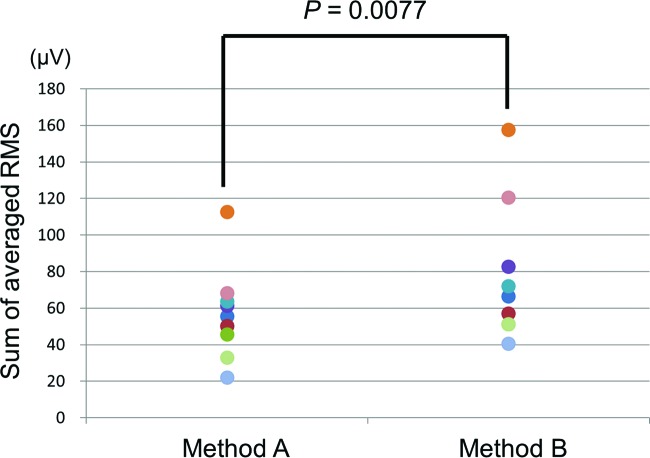
In experienced microsurgeons, method A showed less value of the RMS than method B.

The sums of the averaged RMS values for all subjects are listed in Table 1. Among experienced microsurgeons, method A using the enlarged micro needle holder with pen holding (mean: 57.0 μV) required significantly less forearm muscle activity than method B using a Webster needle holder with scissor holding (mean: 77.7 μV) (*P* = .0077) (Fig 5).

**Figure 6:**
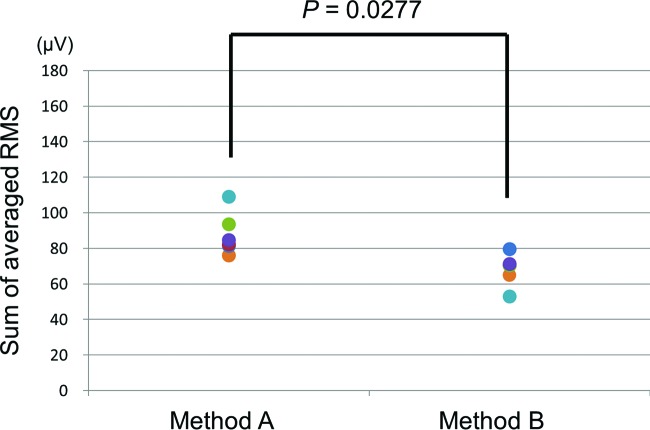
In inexperienced microsurgeons, method B showed less value of the RMS than method A.

In contrast, inexperienced microsurgeons needed more forearm muscle activity for method A (mean: 87.8 μV) than for method B (mean: 68.3 μV) (*P* = .0277) (Fig 6).

## DISCUSSION

According to our results, method A required less forearm muscle movement than method B for experienced microsurgeons, indicating that these surgeons appeared to be already accustomed to using a pen needle holder, regardless of its size. On the contrary, inexperienced microsurgeons required more forearm muscle movement for method A than for method B. This suggested that they were more familiar with the Webster needle holder with scissor holding; in fact, this group had never attempted pen needle holding of any size before the trial.

Because the experienced microsurgeons electromyographically showed less forearm muscle activity for method A, they appeared to favor the intrinsic muscles during fine skin suturing, rather than the extrinsic muscles, as compared with the inexperienced microsurgeons. While the Webster needle holder uses wrist supinating with supinator and extrinsic muscles, the pen needle holder uses finger twisting with intrinsic and extrinsic muscles. Both of the needle holder styles require motion of the extrinsic muscles, which includes the largest tension fraction muscles of the forearm muscles, for twisting the fingers and supinating the wrist. Accordingly, we measured extrinsic muscle activity using sEMG and compared them for each method.

We believed the reason why the experienced microsurgeons used less muscle activity for method A, despite it being their first time to use the enlarged micro needle holder, is that the movements of suturing with the enlarged holder appeared to be identical to those with the micro needle holder, the skill for which had become more or less automatic for this group following long-term practice.^10^

When surgeons learn a new action, they need to pay full attention to carrying it out, but after repetition, the action becomes nearly automatic. This has been described as “motor skill learning.”^11^ Motor skill learning is composed of at least 2 stages: an early (short-term) stage and a late (long-term) stage. Before the acquisition of a skill, correct performance relies on the serial sensorimotor information flow from the association (prefrontal and parietal) cortices to the motor cortices through the premotor cortex. In the early stage of learning, the procedure is acquired predominantly as a spatial sequence by the loop circuit comprising the association cortices and basal ganglia. After long-term practice, however, the procedure is acquired predominantly as a motor sequence dependent on the motor cortices and the middle basal ganglia.^12^ Once a sequence is acquired as a motor sequence with long-term practice, it can be carried out nearly automatically, without attention or working memory.^13^

In our study, it appears that the experienced microsurgeons had already developed the motor sequence for the micro pen-hold method, which enabled them to more effortlessly manipulate the enlarged pen-hold task and save muscle movement in fine skin suturing. On the contrary, the inexperienced microsurgeons had just commenced learning of fine skin suturing with the pen needle holder. Therefore, they may have required much more activation of the cortices than did the experienced microsurgeons and understandably used more forearm muscle activity than with their already familiar Webster method.

## CONCLUSIONS

Experienced microsurgeons may be able to reduce forearm muscle movement by finger twisting in fine skin suturing with an enlarged pen needle holder with pen holding as compared with a Webster needle holder with scissor holding. Although this instrument required more muscle activity for inexperienced microsurgeons, they may benefit from this method for fine skin suturing, to develop their internal model of the dynamics for finger twisting later used in their careers for microsurgery. And, this instrument appears to be suited for cleft lip and palate repair and blepharoplasty because of possibly accurate approximation of the cut edges due to delicate finger twisting with less forearm movement.

## Figures and Tables

**Table 1:** Sum of the averaged RMS (μV) of all subjects

		Sum of Averaged RMS (μV)	
		Method A	Method B
Experienced group	Subject 1	55.7	66.3
	Subject 2	50.6	57.0
	Subject 3	45.7	51.3
	Subject 4	61.3	82.7
	Subject 5	63.7	72.0
	Subject 6	112.7	157.7
	Subject 7	22.0	40.7
	Subject 8	68.3	120.5
	Subject 9	33.0	51.3
Inexperienced group	Subject 10	81.3	79.7
	Subject 11	82.3	70.3
	Subject 12	93.7	70.3
	Subject 13	84.7	71.3
	Subject 14	109.0	53.0
	Subject 15	76.0	65.0

Abbreviation: RMS, root mean square.
